# Notes about Some Seasonable Remedies for Prevalent Summer Diseases

**Published:** 1890-08

**Authors:** 


### Selections and Abstracts

### NOTES ABOUT SOME SEASONABLE REMEDIES FOR PREVALENT SUMMER DISEASES.

Among the diseases that are especially prevalent during the hot weather are the intestinal disturbances—diarrhœa, dysentery, intestinal colic, gastric irritability, etc.

The remedies commended for these affections are manifold, and must necessarily vary with the individual conditions. Simplicity of medicinal treatment in these cases is, we believe, practiced by the most intelligent physicians. As easily available, eligible and convenient remedies, when other than dietetic or hygienic measures are desirable, we may mention the following, supplied by us, which have proved efficient in the experience of many physicians:

Chloranodyne, a preparation of much value as a sedative, anodyne and anti-spasmodic in disturbances of the digestive tract incident to summer. The formula is an improvement upon the chlorodyne of J. Collis Brown, M. R. C. S. L., which has long been established in favor abroad.

Among intestinal sedatives tablets of bismuth subcarbonate and subnitrate, antacid tablets, soda-mint tablets, Dover-powder tablets, pepsin and bismuth tablets, arsenite of copper tablets, and pepsinum purum tablets, or pepsin cordial when diarrhœa is dependent on fermentation of undigested food, offer a choice for selection to meet the varying indications present.

Our pepsin cordial presents the ferment in an especially desirable form for administration, being permanent and palatable, as well as possessing in a high degree the proteolytic properties of the gastric juice.

The antiseptic and sedative treatment of intestinal disorders is a deservedly popular one, and in this class of remedies we would remind physicians of the antiseptic yellow oxide of mercury tablets which have proved of so much service in septic forms of dyspepsia, and prophylactic against diarrhœa and dysentery.

For local use mercuric iodide tablets will be found convenient for making solutions of any desired strength, and for purposes of disinfection of excretions, or surroundings, antiseptic liquid or thiocamph may be employed to advantage.

There is another class of remedies, to which we ask especial attention, of much service in inducing emesis in children or adults in which overloading of the stomach or intestines leads to diarrhœa. A most reliable and certain emetic for this condition is normal liquid ipecac (standard two per cent. emetine). This is valuable also in croup incident to the exposure of children to sudden changes of temperature and the out-door life of summer.

For use as an expectorant for coughs and colds, two recent remedies are being largely employed, viz.: cocillana and Goanese ipecac.

Information as to any of these remedies in the form of descriptive circulars and samples for testing when desired, will be furnished to physicians on request.

PARKE, DAVIS & CO.

